# Caldendrin and Calneurons—EF-Hand CaM-Like Calcium Sensors With Unique Features and Specialized Neuronal Functions

**DOI:** 10.3389/fnmol.2019.00016

**Published:** 2019-02-06

**Authors:** Jennifer Mundhenk, Camilla Fusi, Michael R. Kreutz

**Affiliations:** ^1^RG Neuroplasticity, Leibniz Institute for Neurobiology, Magdeburg, Germany; ^2^Leibniz Group “Dendritic Organelles and Synaptic Function”, Center for Molecular Neurobiology, ZMNH, Hamburg, Germany

**Keywords:** golgi, trafficking, dendritic spines, F-actin, structural plasticity, Ca_V_1.2, InsP_3_R

## Abstract

The calmodulin (CaM)-like Ca^2+^-sensor proteins caldendrin, calneuron-1 and -2 are members of the neuronal calcium-binding protein (nCaBP)-family, a family that evolved relatively late during vertebrate evolution. All three proteins are abundant in brain but show a strikingly different subcellular localization. Whereas caldendrin is enriched in the postsynaptic density (PSD), calneuron-1 and -2 accumulate at the trans-Golgi-network (TGN). Caldendrin exhibit a unique bipartite structure with a basic and proline-rich N-terminus while calneurons are the only EF-Hand CaM-like transmembrane proteins. These uncommon structural features come along with highly specialized functions of calneurons in Golgi-to-plasma-membrane trafficking and for caldendrin in actin-remodeling in dendritic spine synapses. In this review article, we will provide a synthesis of available data on the structure and biophysical properties of all three proteins. We will then discuss their cellular function with special emphasis on synaptic neurotransmission. Finally, we will summarize the evidence for a role of these proteins in neuropsychiatric disorders.

## Introduction

Neuronal Ca^2+^-signaling is based on spatio-temporal gradients called Ca^2+^ waves, spikes, transients and puffs. The strict segmentation of such gradients allows for complex signaling events at the micro- and even nanoscale (Berridge et al., 2003). Consequently, the huge variety of Ca^2+^-evoked processes require a highly specialized machinery leading to alterations in cellular functions and Ca^2+^ sensor proteins from the calmodulin (CaM) family are instrumental in this regard.

## The nCaBP-Family of EF-Hand CaM-Like Calcium Sensor Proteins

**Figure 1:**
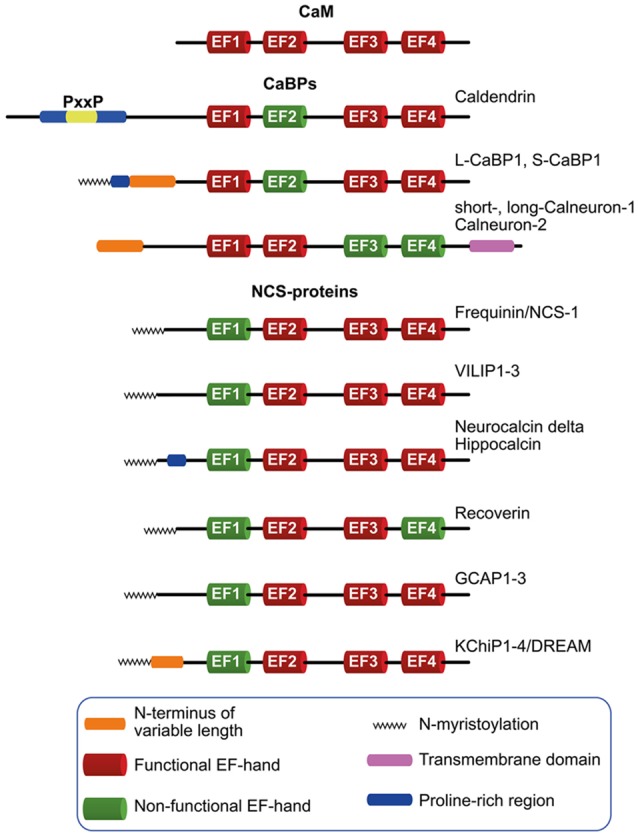
Schematic diagram depicting the neuronal calcium sensor (NCS) and neuronal calcium-binding proteins (nCaBPs) families. Analogous to their ancestral protein calmodulin (CaM), all nCaBPs and NCS-proteins harbor four EF-hands out of which at least one is not capable of binding Ca^2+^ and is therefore referred to as non-functional (green). All NCS family members carry a N-myristoylation (zigzag line) which can be masked in a Ca^2+^-dependent manner in VILIP-proteins, Hippocalcin and Recoverin. Caldendrin, L-CaBP1 and S-CaBP1 share the same EF-hand organization with different N-termini which might define the subcellular distribution of the proteins. The extended N-terminus of caldendrin is characterized by the presence of tandem PxxP motifs. Compared to other nCaBPs and NCS-proteins, calneurons harbor an extension of the C-terminus which is crucial for their targeting to the Golgi-membrane, where they are involved in the regulation of Golgi to plasma membrane trafficking.

The predominant neuronally expressed Ca^2+^-binding proteins of the neuronal calcium sensor (NCS)- and neuronal calcium-binding protein (nCaBP)-family share the same EF-hand organization as their ancestor CaM (Figure 1). In contrast to CaM, however, these proteins exhibit a more restricted and cell type specific expression. They are frequently targeted to subcellular compartments, contain at least one cryptic EF-hand incapable of Ca^2+^-binding, while many of them exhibit a higher global Ca^2+^-binding affinity and a different conformational response upon Ca^2+^-binding than CaM. It is nowadays widely believed that NCS and nCaBP family members are involved in distinct signaling processes and thereby increase the versatility of the Ca^2+^-signaling tool kit (Mikhaylova et al., 2011). NCS proteins are evolutionarily related to Frequenin and, on the basis of their sequences, they have been grouped into five subfamilies (Burgoyne, 2007; Mikhaylova et al., 2011), comprising Frequenin/NCS-1, Recoverin, VILIPs 1–3/Hippocalcin/Neurocalcin delta, GCAP1–3 and the voltage-gated K^+^ (Kv) channel-interacting proteins (KChiPs) 1–4/DREAM (Figure 1).

The nCaBP family consists of six family members, caldendrin, L-CaBP1, S-CaBP1, short- and long-calneuron-1, calneuron-2 (Figure 1). Caldendrin harbors a cryptic EF-hand 2, whereas in calneuron-1 and -2 EF-hands 3 and 4 are non-functional (Figure 1; Mikhaylova et al., 2006; McCue et al., 2010). This excludes interdomain cooperativity in Ca^2+^-binding like it was reported for other CaM-like EF-hand Ca^2+^-sensor proteins (Fefeu et al., 2000; Heidarsson et al., 2013; Ranaghan et al., 2013) including caldendrin (Kiran et al., 2017). Two shorter isoforms arise from alternative splicing of the caldendrin/CaBP1 gene that are denoted L-CaBP1 and S-CaBP1, respectively. Similar to caldendrin, L-CaBP1 and S-CaBP1 harbor a non-functional EF-hand 2 (Haeseleer et al., 2000; Laube et al., 2002). Caldendrin is prominently expressed in brain, whereas S- and L-CaBP1 like all other CaBPs are mainly abundant in retina and the cochlea (Seidenbecher et al., 1998; Haeseleer et al., 2000; Laube et al., 2002; Landwehr et al., 2003; Kim et al., 2014). Thus, in addition to caldendrin, from the nCaBP family only calneuron-1 and -2 are abundant in brain (Hradsky et al., 2015).

## Structural Features and Biophysical Properties of Caldendrin

Caldendrin exhibits a unique bipartite structure with a N-terminal half that contains seven phosphorylation sites with an unusual high proline content (13%) and multiple PxxP motifs (Figure 1). The N-terminus shows no sequence homology to other family members. The shorter splice isoforms share the C-terminus with caldendrin (Figure 1) that resembles the structure of CaM (Figure 1). In contrast to CaM, however, the first EF-hand motif will bind both Mg^2+^ and Ca^2+^ while the second EF-hand is not capable of Ca^2+^ binding. The global affinity of Ca^2+^ and Mg^2+^ binding was found to be in the low μM range (Wingard et al., 2005; Reddy et al., 2014). Since the Mg^2+^-binding affinity is clearly higher than in S-CaBP1 it is likely that the extended N-terminus will impact Mg^2+^-binding to the first EF-hand (Wingard et al., 2005; Reddy et al., 2014). Several lines of evidence suggest complex intra- and intermolecular interactions of caldendrin (Reddy et al., 2014). However, the protein shows very little change conformational change as evidenced by alterations in surface hydrophobicity and secondary as well as tertiary structure upon Ca^2+^-binding to the Mg^2+^-saturated protein (Reddy et al., 2014). Caldendrin is in the C-terminal part the closest homolog of CaM in brain and shares with its ancestor a flexible linker region between both EF-hand domains. But in case of caldendrin insertion of four additional amino acids will allow for a more flexible orientation of both domains that might allow for binding to sterically more complex target interactions (Seidenbecher et al., 1998; Haeseleer et al., 2000; Laube et al., 2002). It was shown that the folding of the EF-hand domains can occur independently and in any order in CaM and probably also all other nCaBP. Unfortunately, still very little is known about the structure-function relationships in relation to ion binding properties of this sensor. Although NMR and crystal structures have been reported for the shorter isoforms (Li et al., 2009; Findeisen and Minor, 2010), the unique N-terminal region and reports pointing towards functional differences between caldendrin and the shorter splice isoforms asks for an independent analysis of their function (Tippens and Lee, 2007; Findeisen and Minor, 2010). Along these lines the rather complex inter- and intramolecular interactions in Caldendrin that are regulated by Ca^2+^-binding, the exceptionally high Mg^2+^- and the relatively low Ca^2+^-binding affinity, the rigid first EF-hand domain and the rather modest changes in confirmation upon binding of Ca^2+^ make it plausible that the association with binding partners might follow different molecular principles in comparison to other neuronal Ca^2+^-sensors.

## The Caldendrin Interactome and Cellular Functions of Caldendrin

Caldendrin is enriched at synapses and in the somatodendritic compartment of pyramidal neurons in cortex and hippocampus (Seidenbecher et al., 1998; Bernstein et al., 2007). Synaptic [Ca^2+^]_i_ levels can be increased by activation of L-type voltage activated Ca^2+^ channels like Ca_V_1.2, N-methyl-D-aspartate receptors (NMDARs) or Inositol trisphosphate receptors (InsP3Rs; Sala and Segal, 2014). It is well established that the activity of InsP3Rs is regulated by CaM (Kasri et al., 2002), however, several reports also indicate that CaBP1, can modulate the activity of InsP3Rs (Haynes et al., 2004; Kasri et al., 2004; Li et al., 2009, 2013). Of note, two binding sites for CaBP1 were identified in InsP_3_Rs and a high affinity interaction requires self-association and a close proximity of both binding domains (Li et al., 2009). Although binding was originally described in contrast to those of CaM to be Ca^2+^-independent, it turned out that Ca^2+^-binding to EF-hands-3 and -4 clearly enhances binding affinity and strengthens the interaction (Li et al., 2009). In addition, it was reported that phosphorylation of CaBP1 by casein kinase II promotes the association (Kasri et al., 2004). In functional terms CaBP1 binding reduces InsP_3_-triggered [Ca^2+^]_i_ release (Haynes et al., 2004; Kasri et al., 2004). Based on NMR studies, it was postulated that a cluster of hydrophobic residues in the C-terminal domain of CaBP1 interact with a complementary cluster of hydrophobic residues in the β-trefold domain of InsP_3_R, trapping the channel in a closed conformation (Li et al., 2013). Upon binding of Ca^2+^ the affinity of CaBP1 was found to be increased for InsP_3_R, locking the channel in the closed state (Li et al., 2009, 2013). Importantly, Li et al. (2009) could show that binding of CaBP1 to InsP_3_R is much stronger than those of CaM to InsP_3_Rs, indicating that the former interaction will easily dominate and is more significant for InsP_3_R function and given the abundance of Caldendrin in spines it will clearly outcompete CaM. Furthermore, the low expression of CaBP1 in the hippocampus makes it likely that caldendrin is the Ca^2+^ sensor for regulating hippocampal InsP_3_Rs and might thereby play an important role in synapto-dendritic Ca^2+^ signaling as a prominent inhibitor of InsP_3_Rs in the hippocampus, a prediction that can be easily tested now due to the existence of caldendrin/CaBP1 knockout mice models (Kim et al., 2014; Mikhaylova et al., 2018; Yang et al., 2018).

Several reports indicate that the association the association of caldendrin/CaBP1 with the α1-C subunit of L-type voltage-dependent Ca^2+^ channels (Ca_V_1.2) is important for its physiological function (Zhou et al., 2004, 2005; Tippens and Lee, 2007; Findeisen and Minor, 2010; Oz et al., 2011, 2013). Caldendrin/CaBP1 reduce the Ca^2+^ dependent inactivation (CDI) of Ca_V_1.2 L-type voltage gated Ca^2+^ channels and thereby facilitate Ca^2+^ currents (Zhou et al., 2004, 2005; Tippens and Lee, 2007; Oz et al., 2011, 2013). In contrast, binding of CaM reportedly results in an inactivation of Ca^2+^ influx and competes with caldendrin/CaBP1 for the same interaction site (Zhou et al., 2004, 2005; Tippens and Lee, 2007). It is plausible that the interaction takes place in the postsynaptic membrane since co-immunoprecipitation of caldendrin and Ca_V_1.2 Ca^2+^ channels was shown with synaptic protein preparations (Zhou et al., 2004, 2005; Tippens and Lee, 2007). The emerging scenario from several studies shows that the regulation of Ca_V_1.2 Ca^2+^ channels by caldendrin, CaBP1 and CaM is complex and that differential binding will allow for a high degree of fine-tuning of synapto-dendritic Ca^2+^ signaling. All three sensors interact with an IQ-domain that is part of the C-terminal cytoplasmic domain of the α1-subunit of Ca_V_1.2. In addition, CaBP1 binds to a second region in the N-terminus and this interaction is in contrast to those of CaM Ca^2+^-independent (Zhou et al., 2005; Dick et al., 2008). Of note, the presence of this domain is essential for inactivation of Ca_V_1.2 Ca^2+^ currents by Caldendrin, but not the CDI mediated by CaM (Zhou et al., 2004). Interestingly, CaBP1 seems to more efficiently inhibit inactivation of Ca_V_1.2 channels than caldendrin (Zhou et al., 2005; Tippens and Lee, 2007). Whereas caldendrin displaces CaM and CaBP1 from the C-terminal IQ-domain it does not interact with the N-terminus of Ca_V_1.2 (Tippens and Lee, 2007). It is therefore likely that the functionally different roles of CaM, CaBP1 and caldendrin are mediated by association to two different domains with different Ca^2+^ requirements.

Other interactions have been studied in much less detail. In functional terms a role in synapse-to-nucleus communication has been ascribed to caldendrin by preventing the nuclear import of the synapto-nuclear messenger Jacob (Dieterich et al., 2008). Caldendrin/CaBP1 knockout mice show a rapid depression at inhibitory presynaptic sites that is related to binding and inactivation of Ca_V_2.1 calcium channels in control of short-term synaptic plasticity (Lee et al., 2002; Few et al., 2011; Leal et al., 2012; Nanou et al., 2018). Other potential binding partners where a cellular and in particular synaptic function is less well investigated include light chain 3 (Seidenbecher et al., 2004), myo1c (Tang et al., 2007), recoverin (Fries et al., 2010), metabotropic glutamate receptors (Nakajima, 2011), AKAP79/150 (Seeger et al., 2012) and Ca_V_1.3 calcium channels (Findeisen et al., 2013; Yang et al., 2018).

## A Specific Synaptic Function of Caldendrin

Interestingly, caldendrin is highly enriched in the postsynaptic density (PSD) of spine synapses, which is very uncommon for a CaM-like Ca^2+^-sensor protein (Seidenbecher et al., 1998; Laube et al., 2002). Dendritic spines are considered as microcompartments of Ca^2+^ signaling (Raghuram et al., 2012) with exceptionally fast Ca^2+^ decay times, much faster than in dendrites (Cornelisse et al., 2007). The presence of “fast” Ca^2+^ buffers are thought to mediate the fast decay of Ca^2+^-transients in spines immediately following influx through Ca^2+^ channels or release from Ca^2+^ stores (Keller et al., 2008). Fast buffers are the first to intercept free Ca^2+^ in the spine. Surprisingly, it was found utilizing UV flash photolysis of DM-nitrophen caged Ca^2+^ that CaM binds Ca^2+^ with a very high on-rate at the N-terminal lobe, even faster than those of classical known calcium buffers like calbindin (Faas et al., 2011). In conclusion, CaM, due to high abundance, its ubiquitous expression and the fast Ca^2+^-binding capacity, has to be considered as the principal buffer in pyramidal neurons. In addition numerous Ca^2+^-dependent targets have been identified which in regulate different aspects of cellular function. In light of these arguments the simple question arises how members of the NCS and nCaBP family operate as calcium sensors in the presence of CaM? Or in other terms will CaM-like sensors even have a chance to compete with CaM for Ca^2+^ binding in spines?

In a recently published study we addressed this question systematically (Mikhaylova et al., 2018). We first found that caldendrin has a similar on rate like CaM for Ca^2+^-binding and can therefore by abundance and properties easily compete with CaM in spines (Mikhaylova et al., 2018). Alterations in synaptic strength require an intimate link between functional and structural plasticity. The latter one is based on the unique cytoskeletal organization of differentially arranged actin filaments. Compartmentalization of calcium-dependent plasticity allows for rapid actin remodeling and in a landmark study, Bosch et al. (2014) addressed the question of structural and molecular remodeling of dendritic spines following the induction of long-term potentiation (LTP). LTP induction results in rapid cofilin-dependent severing of filamentous actin and a massive increase in actin remodeling proteins, which is then followed by a stabilization phase where different F-actin stabilizing and capping proteins gradually increase and only at this later stage reorganization of the PSD comes into play. What is still unclear is how a minimal stable pool of branched actin that is essential for remodeling of F-actin is maintained in spines undergoing plasticity. In fact, molecular machineries coupling local and rapid synaptic elevation of [Ca^2+^]_i_ to actin remodeling in the initial reorganization phase are still unknown. Finally, the role of spatially segregated spinous F-actin pools in transition from short-term to long-term synaptic potentiation was unclear.

**Figure 2:**
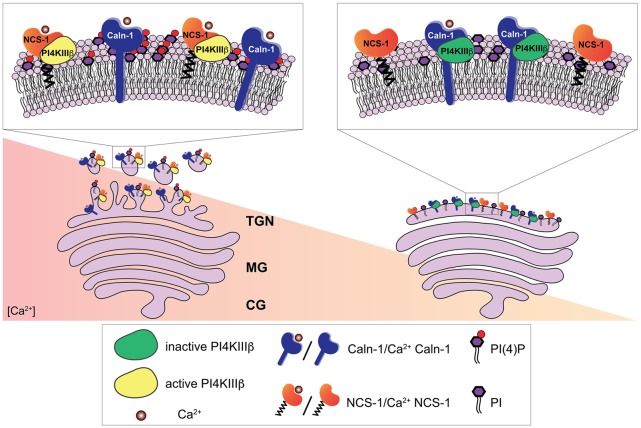
Ca^2+^-dependent regulation of the phosphatidylinositol 4-kinase IIIβ (PI4KIIIβ) by calneuron-1 (Caln-1) and NCS-1 at the trans-Golgi network (TGN). The PI4KIIIβ-catalyzed synthesis of phosphatidylinositol 4-phosphate (PI(4)P) from phosphatidylinositol (PI) at the TGN membrane is a key step in the process of vesicle budding for TGN to plasma membrane trafficking. Ca^2+^-bound calneuron-1 inhibits the activity of PI4KIIIβ at basal intracellular Ca^2+^ concentrations, [Ca^2+^], (right). Middle and high Ca^2+^ concentration (left) can lead to a fully Ca^2+^-bound NCS-1 protein, which then can replace calneuron-1 from PI4KIIIβ thus leading to a strong increase in the production of PI(4)P and vesicle membrane budding.

We could show that caldendrin activates the actin-binding protein cortactin in dendritic spines and thereby stabilizes a synaptic pool of branched F-actin that is essential for the maintenance of LTP (Mikhaylova et al., 2018). We found that steep elevations in spinous [Ca^2+^]_i_ disrupt an intramolecular interaction of caldendrin that hinders access to a series of PxxP-motifs. Opening of the intramolecular interaction results in a rapid association with the SH3 domain of cortactin (Figure 2). A fast on and a slow off rate of binding keep cortactin in an active, F-actin-stabilizing conformation. In this conformation the protein protects a minimal stable spinous F-actin pool against cofilin-induced severing and primes cortactin for sequential binding to N-WASP/Arp2/3 complex in vicinity of the PSD. Caldendrin gene knockout or protein knockdown result in higher actin turnover caused by loss of stable pool of actin filaments and a disordered topology of spinous F-actin which lead to a loss of spatial segregation of F-actin nanodomains, defects in structural spine plasticity, LTP and hippocampus dependent learning (Mikhaylova et al., 2018). We think that caldendrin directly couples elevation of [Ca^2+^]_i_ to the stabilization of actin branches in a very early step of temporary gating for F-actin remodeling in dendritic spines and thus controls the life time of functionally different F-actin pools during the reorganization phase (Figure 2). This mechanism is an essential component of structural plasticity. Accordingly, it was reported in a recent study (Yang et al., 2018) that caldendrin knock-out mice exhibit deficits in spatial learning and memory and also fear-related memories. Surprisingly, it was also found that adult neurogenesis in the hippocampus is severely impaired in knock-out animals by yet unknown mechanisms (Yang et al., 2018). It is likely that this impairment will also contribute to learning and memory deficits in these mice.

## The Transmembrane nCaBP Family Members Calneuron-1 and -2

Calneuron-1 and -2 (also called CaBP8 and CaBP7; McCue et al., 2010) configure a separate subfamily within the nCaBP family (Mikhaylova et al., 2006, 2011). In contrast to other NCS- and nCaBP proteins they harbor a non-functional C-terminal EF-hand domain (Figure 1; Wu et al., 2001; Mikhaylova et al., 2006; McCue et al., 2010). Both calneurons show 64% identity at the amino acid level when compared to each other whereas less than 30% identity exists in comparison to CaBPs and caldendrin (Mikhaylova et al., 2006; McCue et al., 2010). The loops of the functional EF-hands 1 and 2 are almost identical (Mikhaylova et al., 2006) and also the loops of EF-hand 3 exhibit high similarity. Calneurons have the highest Ca^2+^-affinity of all identified CaM-like EF-hand Ca^2+^-sensors with a global dissociation constant of 230 and 180 nM for calneuron-1 and -2, respectively (Mikhaylova et al., 2009). However, these affinities were measured for proteins without membrane insertion, which might have obscured the outcome of these measurements. Apart from differences within the EF-hand domains calneurons harbor in addition an extension of the C-terminus compared to other nCaBPs and NCS-proteins, which is crucial for the targeting of these proteins to the Golgi-membrane (McCue et al., 2010; Hradsky et al., 2011), where they are involved in the regulation of Golgi to plasma membrane trafficking (Mikhaylova et al., 2009). Being almost identical in both proteins the C-terminus contains a highly hydrophobic stretch, which configures a transmembrane domain (TMD; Hradsky et al., 2011; McCue et al., 2011). Since both calneurons do not harbor a N-terminal myristoylation motif, transmembrane insertion of these proteins would explain their strong membranal association as compared to other neuronal Ca^2+^-sensors like caldendrin. Of note they are the only transmembrane CaM-like calcium sensors and this points to a probably unique role in the neuronal Ca^2+^-sensing toolkit.

## Calneuron-1 and -2 Are Tail Anchored Proteins That Regulate Golgi-to-Plasma Membrane Trafficking

The C-terminal position of the transmembrane segment indicates that calneurons belong to the group of tail-anchored proteins, which have to undergo post-translational insertion (Borgese et al., 2003, 2007). It turned out that calneurons are indeed non-classical type II tail-anchored proteins and their posttranslational insertion into the ER membrane *via* an association of the TMD with the TRC40/Asna1 chaperone complex was demonstrated (Hradsky et al., 2011). Their tight association with the trans-Golgi-network (TGN) might be explained by the length of the TMD and phosphatidylinositol 4-phosphate (PI(4)P) lipid binding (Hradsky et al., 2011). Self-association *in vitro* and *in vivo* occurs *via* the TMD and EF-hand containing N-terminus. Despite the fact that dimerization will hinder TRC40/Asna1 binding and in consequence membrane insertion, evidence for the existence of a cytosolic non-membrane associated pool of calneurons is currently lacking and dimerization was only found for membrane inserted protein (Hradsky et al., 2011). This almost exclusive and unique association with membranes of the secretory pathway indicates a probably highly specific function with a limited number of target interactions.

**Figure 3:**
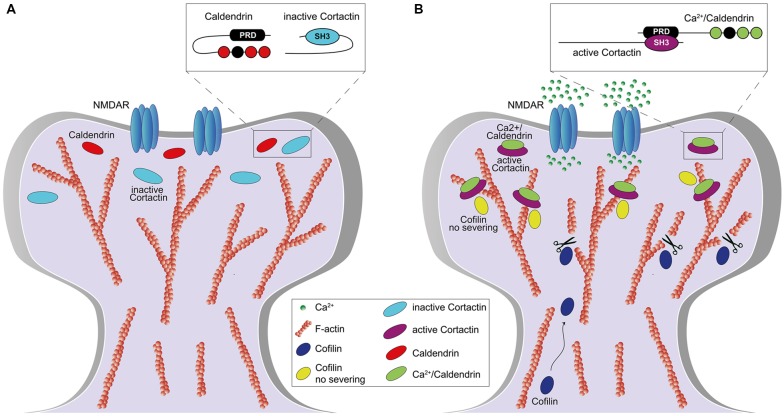
Role of caldendrin and cortactin in the stabilization of actin filaments in the initial phase of synaptic potentiation. **(A)** Under basal condition, caldendrin and cortactin are kept in an inactive state which prevents the exposure of caldendrin N-terminal proline-rich domain (PRD) and cortactin SH3-domain (**A**, inset). **(B)** Synaptic stimulation, *via* N-methyl-D-aspartate receptor (NMDAR) activation, leads to an influx of Ca^2+^ which is bound by caldendrin. Ca^2+^ binding results in a conformational change of caldendrin with subsequent exposure of the PRD which associates with the SH3-domain of cortactin, keeping cortactin in an active and open conformation (**B**, inset). Caldendrin-cortactin complex is then anchored to F-actin and prevents cofilin-mediated filament severing. At the same time, a rate of caldendrin-cortactin-free F-actin filaments are severed by cofilin, whose levels increase upon NMDARs activation, allowing generation of new filaments and reorganization of the spine.

In functional terms calneurons play an important role at the Golgi-apparatus where they control TGN to plasma membrane trafficking by regulating the activity of phosphatidylinositol 4-OH kinase IIIβ (PI-4KIIIβ; Mikhaylova et al., 2009). PI-4KIIIβ catalyzes local synthesis of phosphoinositides necessary for vesicle budding at the TGN. Calneurons directly bind to PI-4KIIIβ and inhibit the enzyme at low [Ca^2+^] levels (Figure 3; Mikhaylova et al., 2009). With increased [Ca^2+^] levels the inhibition is released and PI4KIIIβ is activated *via* a preferential association with NCS-1. Taken together that data suggest that calneurons establish a [Ca^2+^] threshold for activation of the enzyme (Figure 3; Mikhaylova et al., 2009). Of note, the tight association of calneurons to the Golgi can be even used to target proteins to the TGN (Bera et al., 2016). The TMD of calneuron-2 was employed to develop a plasmid-based expression system called pGolt that has the advantage to fuse other proteins to the extraluminal part. This in turn makes it possible to study protein-protein interactions outside of the Golgi lumen (Bera et al., 2016). An obvious and particularly promising application in neuroscience is to use this Golgi-tracker system for the visualization of Golgi outposts (GOs). In non-neuronal cells the organelles of the secretory pathway have a highly restricted spatial organization. In stark contrast in neuronal cells along with the localization of secretory organelles in the cell soma, numerous discrete and discontinuous structures resembling Golgi cisternae are present along dendrites, which are known as GOs. We next showed with pGolt the presence of Golgi-related organelles in all dendrites of pyramidal neurons in close proximity to endoplasmic reticulum-Golgi intermediate compartment and retromer (Mikhaylova et al., 2016). We found that this Golgi-Satellite secretory system (GS) in dendrites is much more widespread than previously described GOs. Most importantly, this GS contains at least part of the cellular glycosylation machinery but as opposed to GOs lacks many protein components for sorting and organization of Golgi cisternae. Moreover, we realized that a broad spectrum of synaptic transmembrane proteins (including GluA1, GluN1, GluN2B, NCAM and Neuroligin-1) might pass and even recycle through these organelles and that also calneuron-1 is present at GS (Mikhaylova et al., 2016). Collectively the study suggest that GS will enable local glycosylation of proteins, and that these proteins will be subsequently recruited to membranes in spatially confined dendritic segments. It will be interesting to investigate in the future whether calneuron-1 has a similar role at GS like at the TGN. Of note, another report (Rajamanoharan et al., 2015) indicated that calneuron-2 mediated inhibition of PI4KIIIβ is instrumental for cytokinesis in HeLa cells. In this study, it was reported that calneuron-2 associates with lysosomes and regulates their clustering and that PI4KIIIβ plays an important role for normal cytokinesis (Rajamanoharan et al., 2015).

## Other Possible Cellular Functions of Calneurons in Neuronal Ca^2+^-Signaling

Compared to the multitude of CaM protein interactions, the interactome of nCaBPs and here in particular of calneurons is relatively small. In several studies an interaction with G-protein coupled receptors (GPCRs) was reported (Navarro et al., 2014; Angelats et al., 2018; Franco et al., 2018). It is well established in striatal neurons that [Ca^2+^]_i_ impact modulation of A2AR-D2R heteromers that is mediated adenylyl-cyclase and MAP-kinases. Differential modulation is based on the interaction of the heteromer with NCS-1 and calneuron-1 at low and high [Ca^2+^]_i_, respectively (Navarro et al., 2016). The association with both Ca^2+^-binding proteins appears to differentially regulate subsequent allosteric interactions within the A2AR-D2R heteromer. Thus, binding of both proteins configures a unique cellular mechanism to integrate extracellular (adenosine and dopamine) and [Ca^2+^]_i_ signals in order to elicit a specific downstream signaling event (Navarro et al., 2016). In the past years further interactions with either GPCR heteromers or heteromers of GPCR with glutamate receptors were reported (Angelats et al., 2018; Franco et al., 2018) and the emerging picture suggests that: (i) [Ca^2+^]i determines the binding affinity of different sensors; and that (ii) in case of cooperative binding differential intracellular responses can be elicited depending upon binding. What is clearly missing is an understanding of the cellular function of these interactions. This also holds true for work that shows an interaction between calneuron-1 and N-type calcium channels (Shih et al., 2009), an interaction that could play a role in the modulation of action potential firing or neurotransmitter release. Finally, calneuron-1 is highly expressed in aldosterone producing adenoma cells where it regulates the storage of Ca^2+^ into the ER (Kobuke et al., 2017). Overexpression of calneuron-1 leads to an increased Ca^2+^ level in the ER and aldosterone overproduction (Kobuke et al., 2017). A mechanistic explanation for this phenotype, however, is lacking.

## On a Putative Role of Calneuron-1 and Caldendrin in Mental Disorders

The onset of schizophrenia usually takes place in young adulthood between the age of 20–30 (Thompson et al., 2004). It is widely believed that subtle developmental alterations in brain structure and connectivity in the interaction with environmental factors cause schizophrenia in early adulthood. The underlying processes have been also related to modifications of the epigenome. Three relatively recent studies from different groups propose that the human CALN1 is a candidate schizophrenia gene (Li et al., 2015; Xia et al., 2015; Roussos et al., 2016) but it is at present unclear how calneuron-1 function can be related to psychotic behavior. However, it has been shown that alteration in methylation levels on the CALN1 and the AUTS2 gene occur in schizophrenia patients (Wockner et al., 2014). A recently published study (Engmann et al., 2017) shows that the exposure to drugs of abuse, like cocaine, alters epigenetic patterns of certain brain regions due to chromatin modifications. Engmann et al. (2017) found that the methylation of the CALN1 promoter increases the expression of AUTS2, which is another known risk gene for schizophrenia (Zhang et al., 2014), and vice versa. They show that methylation of the AUTS2 or the CALN1 promoter enhances calneuron-1 expression although both genes are not in close proximity to each other. The distance of around 1542 Kb between both genes is bypassed by an epigenetic modification called chromatin looping, which enables regulatory elements such as enhancers, which are located relatively far from the transcription start site, to interact with the associated promoter regions using CCCTC-binding-factors (Sexton et al., 2009; Engmann et al., 2017). Interestingly, a meta-analysis of the genetic association studies came to the conclusion that calneuron-1 mRNA levels might be up-regulated in schizophrenia in the dorsolateral prefrontal cortex (Ohi et al., 2016). A potential mechanism might have been proposed by Engmann et al. (2017), whose results are indicating that an overexpression of CALN1 might lead to an overexpression of AUTS2, a well-known risk gene for schizophrenia, *via* chromatin looping. Alternatively, higher calneuron-1 protein levels might have independently an effect on cellular signaling that is related to psychotic behavior.

Interestingly, caldendrin protein levels are also regulated in human schizophrenia (Bernstein et al., 2007) and in mouse models of drug-induced psychosis (Smalla et al., 2009). Fewer caldendrin-immunopositive neurons are found in the left dorsolateral prefrontal cortex in schizophrenia patients, and it is tempting to speculate that synaptic and/or dendritic Ca^2+^-signaling is altered in schizophrenia due to a redistribution of the protein. Thus, it appears that the remaining pyramidal neurons expressing caldendrin exhibit higher protein levels. In summary, although it is at present unclear how exactly both proteins contribute to the pathophysiology of schizophrenia, the basic characterization of their unique cellular role of calneuron-1 might pave the way to understand how they are involved in schizophrenia.

## Author Contributions

JM, CF, MK wrote the review article. CF designed the figures.

## Conflict of Interest Statement

The authors declare that the research was conducted in the absence of any commercial or financial relationships that could be construed as a potential conflict of interest.
